# Increasing Lifestyle Walking by 3000 Steps per Day Reduces Blood Pressure in Sedentary Older Adults with Hypertension: Results from an e-Health Pilot Study

**DOI:** 10.3390/jcdd10080317

**Published:** 2023-07-27

**Authors:** Elizabeth C. Lefferts, Joseph M. Saavedra, Bong Kil Song, Angelique G. Brellenthin, Linda S. Pescatello, Duck-chul Lee

**Affiliations:** 1 Department of Kinesiology, Iowa State University, Ames, IA 50011, USA; eschro@iastate.edu (E.C.L.); joeysav@iastate.edu (J.M.S.); abrellen@iastate.edu (A.G.B.); 2 Department of Kinesiology, University of Connecticut, Storrs, CT 06269, USA

**Keywords:** steps/day, physical activity, aging, intervention

## Abstract

Increasing daily steps by an additional 3000 steps/day on 5 days/week equates to ~150 min/week of aerobic physical activity to meet the physical activity guidelines; however, its effectiveness for blood pressure control in older adults with hypertension is unknown. A 20-week, single-arm, pilot e-health lifestyle walking intervention was conducted in 21 sedentary older adults (73 ± 5 years old) with hypertension (13 female, 8 male) to investigate the effectiveness of increasing daily steps by an additional 3000 steps/day for blood pressure control. The intervention consisted of two phases, with behavior change assistance provided during the first active phase (weeks 1–10) to help reach step goals and minimal assistance provided during the second self-maintenance phase (weeks 11–20). Nineteen participants (91%) completed both the 10- and 20-week assessments. The participants wore the pedometer for ≥10 h on 97% of the days over 20 weeks. They significantly increased average steps/day from 3899 ± 2198 at baseline to 6512 ± 2633 at 10 weeks and 5567 ± 2587 at 20 weeks. After 20 weeks, both systolic (137 ± 10 to 130 ± 11 mm Hg, *p* < 0.001) and diastolic (81 ± 6 to 77 ± 6 mm Hg, *p* = 0.01) blood pressure improved. The response was consistent in participants with (*n* = 8) and without (*n* = 13) anti-hypertensive medication. The results of our lifestyle walking intervention are encouraging for reducing blood pressure in older adults with hypertension; however, larger randomized, controlled trials need to be performed to confirm these findings.

## 1. Introduction

Approximately eight out of 10 adults 65 years of age or older in the United States are burdened with high blood pressure, one of the leading risk factors for cardiovascular disease morbidity and mortality [1]. Lifestyle modification is often the first line of treatment for high blood pressure management, with increasing physical activity a critical component [2]. Unfortunately, despite the known the benefits of physical activity for health, barriers often exist to performing regular structured physical activity (e.g., gym-based exercise) for older adults [3]. Structured physical activity, however, may not be necessary to reduce blood pressure, as previous studies in sedentary adults have suggested that lifestyle physical activity may be just as effective [4,5]. The most common lifestyle physical activity in older adults is walking, which is accessible, inexpensive, and easy to implement for public health impact [6]. One effective strategy for increasing walking in older adults is the implementation of an e-health intervention (i.e., enhancing health services through electronic devices, the internet, and/or related digital technology) [7]. Thus, using an e-health intervention to increase lifestyle walking may be an effective lifestyle modification to improve blood pressure in older adults with hypertension. However, the number of steps/day that older adults should achieve for health benefits is unclear.

Walking 10,000 steps/day is a common public health goal but may be unrealistic and difficult to achieve in older adults, who traditionally have lower daily step counts [8,9,10,11], averaging 4200 steps/day [12]. Current physical activity guidelines in the United States recommend that all adults should perform at least 150 min per week of moderate to vigorous aerobic physical activity for substantial health benefits [13]. In sedentary older adults, achieving an absolute number of steps/day (e.g., 10,000 steps/day) may therefore be less critical for health improvements compared to increasing the amount of time spent walking relative to baseline levels. Previous research has suggested that using pedometers increases physical activity and elicits significant reductions in blood pressure in the general population [14]. Walking an extra 3000 steps/day on 5 days per week equates to approximately 150 min of physical activity [15] and has previously been used as a target to meet the physical activity guidelines [16]. Interestingly, previous studies have suggested that increasing steps/day to meet the physical activity guidelines, irrespective of the absolute number of total steps (e.g., 10,000 steps/day), can confer health benefits such as reducing blood pressure in postmenopausal women [17] and middle-aged women with obesity [18]. The effectiveness of an e-health step intervention to increase lifestyle walking by an extra 3000 steps/day on 5 days per week for blood pressure control in older adults with hypertension, however, is currently unknown.

The primary purpose of this pilot study was to test the feasibility and effectiveness of an e-health lifestyle walking intervention for blood pressure control in sedentary older adults (≥65 years old) with hypertension (systolic blood pressure 130–159 mm Hg and/or diastolic blood pressure 80–99 mm Hg; or currently taking anti-hypertensive medication). We specifically examined whether an extra 3000 steps/day on 5 days/week for 10 weeks (phase 1: active intervention) reduced blood pressure in sedentary older adults with hypertension. Further, the maintenance of a physical activity program is often difficult, with most older adults returning to inactive habits following an intervention [19]. Thus, the second aim of this pilot study was to determine whether sedentary older adults with hypertension could independently maintain an extra 3000 steps/day on 5 days/week with minimal research personnel contact for an additional 10 weeks (phase 2: self-maintenance). We hypothesized the lifestyle walking intervention would reduce blood pressure in sedentary older adults with hypertension and that the participants would be able to maintain the increased walking throughout the entire 20-week intervention.

## 2. Materials and Methods

### 2.1. Study Participants

We recruited sedentary older adults at least 65 years of age (range 66–83 years) with hypertension (systolic blood pressure 130–159 mm Hg and/or diastolic blood pressure 80–99 mm Hg; or currently taking anti-hypertensive medication) who were overweight or obese (body mass index [BMI] 25–40 kg/m^2^) and therefore at increased risk for developing cardiovascular disease. Participants were recruited via e-mail in September and October 2020 during the COVID-19 pandemic from the Physical Activity and Aging Study (PAAS), an ongoing prospective cohort study of more than 900 older adults in central Iowa, designed to investigate the associations of physical activity and fitness with chronic disease prevention and longevity in older adults. Individuals who responded to the e-mail with study interest were initially screened via phone calls and were excluded for a history of heart attack, stroke, or cancer diagnosis within the previous 6 months; any significant mobility limitation impacting walking; self-reported BMI < 25 kg/m^2^ or >40 kg/m^2^; current smoking; or self-reported meeting of the current aerobic physical activity guidelines of 150 min per week over the previous 3 months following the International Physical Activity Questionnaire (IPAQ) [20]. After the telephone screening, eligible participants were invited to complete the baseline assessment to confirm eligibility based on blood pressure, average steps/day, and body mass index. Participants taking anti-hypertensive medication were not excluded from the study but were asked to maintain their medication (dose and frequency) throughout the course of the study. Eight participants were taking anti-hypertensive medications: two were taking angiotensin II receptor antagonists, one was taking a beta blocker, one was taking a calcium channel blocker, one was taking a calcium channel blocker and an angiotensin II receptor antagonist, two were taking a beta blocker and a combination angiotensin II receptor antagonist/diuretic, and one was taking an ACE inhibitor and diuretic. If participants performed, on average, >8000 steps/day, they were considered active and were excluded from the intervention. Previous studies have suggested that individuals with >8000 steps/day are considered to be active and likely already receive the cardiovascular health benefits of physical activity [16,21]. This study was performed according to the Declaration of Helsinki, and the study protocol was approved by the university’s Institutional Review Board (20-375-00). All participants provided written informed consent.

### 2.2. Study Design

**Figure 1:**
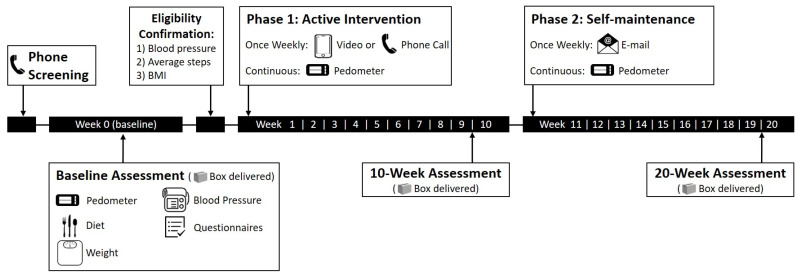
Study design.

This investigation utilized a single-arm, within-participant design, completing assessments at baseline, 10 weeks, and 20 weeks during the intervention period (Figure 1). Eligible participants following the phone screen were assigned a single health coach for all communication and assessment instruction throughout the intervention. The e-health intervention and assessments were all conducted using digital (e.g., video call via provided iPad) physical activity coaching and tele-counseling (e.g., via telephone), which was considered to be a practical and scalable approach for a future large-scale intervention.

All assessments (baseline, 10 weeks, 20 weeks) occurred over 8 days, and the baseline assessment served to confirm study eligibility based on average daily steps, blood pressure, and body mass index. Prior to each assessment, research personnel delivered a box of equipment to participants (e.g., pedometer, blood pressure monitor, weight scale, questionnaires) for at-home self-assessments. On the first day, the participants had an individual virtual meeting with their assigned health coach to complete the medical history questionnaire and learn how to use all equipment appropriately. Instructions also included paper handouts and a live video demonstration by the health coach. On day 9, research personnel retrieved all research equipment, questionnaires, and logs from the participants.

#### 2.2.1. Assessments

The participants were instructed to measure blood pressure and heart rate on days 2 and 3 in both the morning and evening using the provided automated device (Omron HEM-907XL). Morning blood pressures were to be measured before eating breakfast and taking medications, whereas evening measures were performed before the evening meal. The participants were instructed to rest for at least 10 min in a seated position with no constrictive clothing on their left upper arm, legs uncrossed, back and arm supported, and no distractions. The participants performed three measurements at each time point with at least 2 min of rest between measures. In total, the participants recorded 12 blood pressure and heart rate measurements on a provided blood pressure log. Blood pressure and heart rate were recorded at the same time of day as baseline at the 10- and 20-week assessments to avoid the impact of diurnal variation. All values were averaged at each time point for subsequent analysis, following the American Heart Association guidelines [22].

Body weight was assessed in the morning wearing minimal clothing after using the restroom and before assessing blood pressure for 2 days using a standard scale (Eat Smart Precision Plus Digital Bathroom Scale, Oak Brook, IL, USA). The participants recorded their weight on the provided log, and the 2-day average was used for subsequent analysis. Height was obtained from the most recent measurement (average 15 ± 4 months prior) as part of their participation in the PAAS parent study [23]. Body mass index (BMI) was subsequently calculated as weight (kg) divided by height (m) squared.

On day 6, a 24-h dietary recall was completed over the phone with research personnel using the Automated Self-Administered 24-h Recall (ASA24^®^), version 2020, a web-based system developed by the National Cancer Institute, Bethesda, MD, USA [24]. To reduce participant confusion, research personnel assisted the participants with the completion of the dietary recall by verbally asking the participants to recall all foods and beverages consumed on the previous day from midnight to midnight, instead of using the self-administration feature. The participants provided information about time of day, food preparation, and portion size. The nutrition data were automatically analyzed to provide total kilocalories and individual levels of protein, fat, carbohydrates, fiber, and sodium and were validated against standardized interviewer-administered 24-h recalls [25]. 

The Self-Report Habit Index (SRHI) questionnaire was completed to determine the overall strength of walking as a habit [26]. The questionnaire consisted of 12 items relating to walking, and responses to each item were ranked on a 7-point Likert scale [1 = completely disagree (not a habit); 7 = completely agree (strong habit)]. The 12 items were averaged for use in analysis, with a higher score indicative of a stronger habit. The SRHI has high test–retest reliability (*r* = 0.91) and convergent validity (*r* = 0.58) [26].

The social cognitive determinants of physical activity were assessed via two questionnaires adapted from Caudroit et al. [27] and Parschau et al. [28] to specifically reflect our additional 3000 step/day intervention (see Supplementary File S1). The Exercise Motivation Questionnaire was a 12-item survey completed at baseline that captured risk perception, outcome expectancies, motivational self-efficacy, and intentions before starting the intervention. The Exercise Action Questionnaire was a 12-item survey completed at 10 and 20 weeks, measuring action planning, coping planning, maintenance self-efficacy, and recovery self-efficacy during the intervention. Both questionnaires had 7-point Likert scales ranging from very unlikely/not at all true/completely disagree to very likely/definitely true/completely agree, similar to prior studies [27,28]. Responses on each questionnaire were averaged to create an overall motivation (baseline) and action score (10 weeks, 20 weeks), which are two phases of behavior change that reflect the transition from initial intentions and motivations surrounding a new behavior to the actual performance of a new behavior [29]. The higher the score (i.e., closer to 7), the greater the motivation or action perceived by the participant. 

#### 2.2.2. Intervention

Baseline steps/day were measured on days 2 through 8 of the baseline assessment using a sealed (i.e., steps not visible to the participant, step count total blocked using opaque tape) tri-axial accelerometer-based pedometer (Omron HJ-321, Lake Forest, IL, USA) [30]. The participants were instructed to wear the sealed pedometer attached to the hip or inside their pocket, removing it only during water-based activities (e.g., bathing, swimming) or sleeping, to obtain unbiased baseline steps. The pedometer has a 7-day memory and automatically resets at midnight. The participants self-reported wear time on a paper pedometer log. Research personnel retrieved the sealed pedometer and pedometer log on day 9 of the baseline assessment to record the previous 7 days of steps. Baseline steps/day were considered valid if participants wore their pedometer for at least 10 h on at least 5 of the previous 7 days. Baseline steps/day were subsequently determined by averaging the daily steps from at least 5 days with a wear time of at least 10 h. The pedometer also automatically records aerobic steps, which are steps taken at a pace ≥ 60 steps/min during continuous walking of ≥10 min without interruption. Aerobic steps/day were calculated by averaging the aerobic steps from all valid days [31]. 

If participants averaged <8000 steps/day, they were enrolled in the 20-week e-health lifestyle walking step intervention. The participants were prescribed a step count target equivalent to the increase in their daily lifestyle (baseline) step count by 3000 steps/day on at least 5 days per week for 20 weeks. For the intervention, research personnel provided an unsealed (i.e., digital display fully visible) pedometer to wear daily and a pedometer log to record steps and daily wear time. The participants were not instructed to achieve their step goals following strict requirements for time or intensity of walking, but they could accumulate steps throughout the day in any manner that fit their lifestyle. To reduce risk of injury, the participants were asked to only increase by 3000 steps/day on 3 days of the week for the first week, with at least 5 days/week requested from week 2 to week 20. Other than increasing walking behavior, the participants were asked to maintain their usual lifestyle physical activity and dietary habits. Average steps/day were calculated by week throughout the intervention and were determined by averaging the daily steps from all days with at least 10 h of wear time, regardless of whether or not the step goal was achieved on that day. 

The participants received a paper handout to put in a visible place around their homes to promote different strategies to increase steps/day (see Supplementary File S2). During the first 10 weeks of the intervention, the participants also individually received scheduled, weekly video calls or telephone calls from their health coach to answer any study-related questions, collect numbers of steps recorded for the last week, and assist with behavior change techniques to meet their goals. While the health coach incorporated behavior change techniques emphasized in the Health Action Process Approach (HAPA) Theory, such as social support, goal setting, problem solving, and habit formation, to support all participants in meeting their step goals, the participants were originally randomized to two different deliveries of the HAPA theory (trial retrospectively registered at ClinicalTrials.gov, NCT05433233) [29]. In addition to the techniques already listed, one group received an additional 10–15 min of structured conversation about different behavioral change techniques during the first 10 weeks (*n* = 10). However, both groups significantly and similarly increased average steps/day and reduced blood pressure over the 20 weeks (see Supplementary File S3), likely due to all participants receiving substantial behavior change counseling (e.g., goal setting). All data were therefore combined for this study.

For weeks 11 through 20, research personnel did not assist with managing the behavior change, and all participants independently recorded steps using the pedometer log. Research personnel had minimal contact, using weekly e-mails to obtain step information from the participants during this time. 

Adherence was assessed separately for adherence to wearing the pedometer and adherence to the intervention. Adherence to the measurement protocol (i.e., valid reporting of steps) was determined by assessing the percentage of days of the 140 total days on which a participant wore the pedometer for at least 10 h over the 20-week intervention (e.g., 7 days × 20 weeks). Adherence to the intervention (i.e., meeting the step goal) was determined by assessing the number of days out of the prescribed 50 days on which the participants wore their pedometer for at least 10 h and achieved their step goal during each 10-week period (e.g., 5 days × 10 weeks). 

### 2.3. Statistical Analysis

Data are presented as means and standard deviations or medians and interquartile ranges when non-normally distributed. Normality was assessed via the Shapiro–Wilk test. Intention-to-treat analyses were performed using the last observation carried forward. The analyses were also repeated using only individuals with complete data (data at all 3 assessments, *n* = 19) to confirm results. A linear mixed-effects model including time (repeated) was used to assess the change in the primary outcome, blood pressure, as well as all other secondary outcome variables, after adjusting for sex and baseline values. Simple Hedges g_av_ effect sizes (g_av_) for dependent samples were calculated between two time points [32], with 0.2, 0.5, and 0.8 suggesting small, medium, and large effects, respectively. Correlation analyses were performed to examine the relationship between baseline blood pressure and changes in blood pressure during the intervention, as well as between exercise motivation and action with changes in steps/day. Exploratory analyses were performed considering sex as a biological variable within the primary outcomes; however, no significant interaction effects were detected (data not shown). Statistical analyses were performed using SAS software (SAS Institute, Cary, NC, USA). All *p*-values were two-sided, with an a-priori α-level of 0.05 deemed significant.

## 3. Results

A total of 59 individuals completed the phone screen, with 16 ineligible for the baseline assessment due to being too active (*n* = 15) or having cancer within previous 6 months (*n* = 1). Of the 43 eligible for the baseline assessment, 6 dropped out prior to starting (time commitment, *n* = 3; COVID, *n* = 3) resulting in 37 participants who completed the baseline assessment. Of those 37, 16 participants were ineligible based on blood pressure < 130/80 mm Hg (*n* = 9), age (*n* = 1), other conflicting study enrollment (*n* = 1), and BMI (*n* = 5), resulting in 21 participants. One participant discontinued the intervention before the 10-week assessment due to a COVID-19 diagnosis, and 1 participant died before the 20-week assessment, which was unrelated to the intervention. Thus, 20 participants completed the 10-week assessment, whereas 19 completed the 20-week assessment. Baseline characteristics are presented in Table 1. Body weight and BMI did not significantly change throughout the intervention (*p* > 0.05, Table 2). Additionally, no significant dietary changes were observed for total kilocalories, protein, fat, carbohydrates, fiber, or sodium (*p* > 0.05, Table 2).

**Figure 2:**
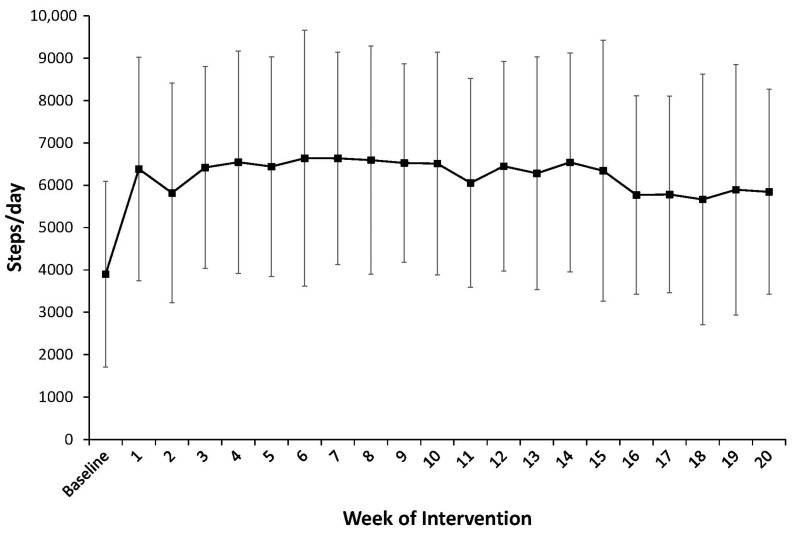
Average steps per day across 20 weeks of lifestyle walking intervention. Steps were significantly higher than baseline during all weeks of intervention (*p* < 0.05 for each). At baseline, 10 weeks, and 20 weeks, average daily steps were 3899 ± 2198, 6512 ± 2633, and 5567 ± 2587 steps/day, respectively. Error bars indicate standard deviations.

The participants were adherent to the study protocol with a median (IQR) adherence of 97% (89%, 99%), indicating that they wore the pedometer for at least 10 h on 97% of the days of the 20-week intervention. Weekly average steps/day are shown in Figure 2. At baseline, the participants averaged 3899 ± 2198 steps/day. Average daily steps increased by 2760 ± 1372 steps/day, resulting in 6512 ± 2633 total steps/day (*p* < 0.001, g_av_ = 1.09), an average increase of 101 ± 86% at 10 weeks. Steps/day remained significantly higher than baseline at 20 weeks (*p* < 0.001, g_av_ = 0.84), with an average increase of 2103 ± 1541 steps/day, resulting in 5567 ± 2587 total steps/day, an average increase of 77 ± 97%. Aerobic steps/day also increased (*p* < 0.001) from 751 ± 1047 steps/day to 3277 ± 576 and 2638 ± 1909 steps/day at 10 and 20 weeks, respectively. Throughout the intervention, the participants were asked to meet their step goals on 5 days/week over each 10-week phase, resulting in 50 total days in each phase to meet their step goals. On average, the participants met their step goals on 43 ± 14 days (86%) and 40 ± 20 days (80%) during weeks 1–10 and 11–20, respectively. 

**Figure 3:**
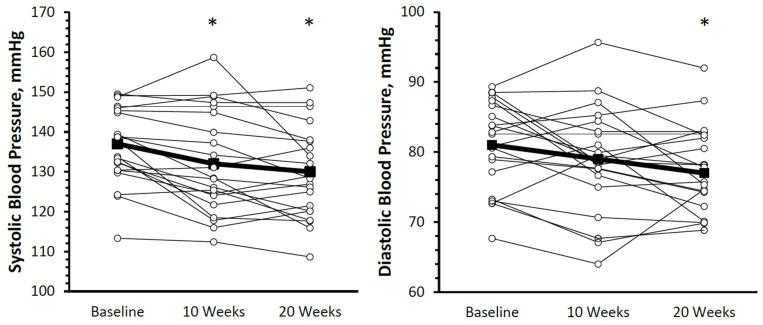
Individual (○) and mean (■) changes in blood pressure. At baseline, 10 weeks, and 20 weeks, systolic and diastolic blood pressure decreased from 137 ± 10 to 132 ± 13 to 130 ± 11 mm Hg and 81 ± 6 to 79 ± 8 to 77 ± 6 mm Hg, respectively. * mean significantly different from baseline, *p* < 0.05.

**Figure 4:**
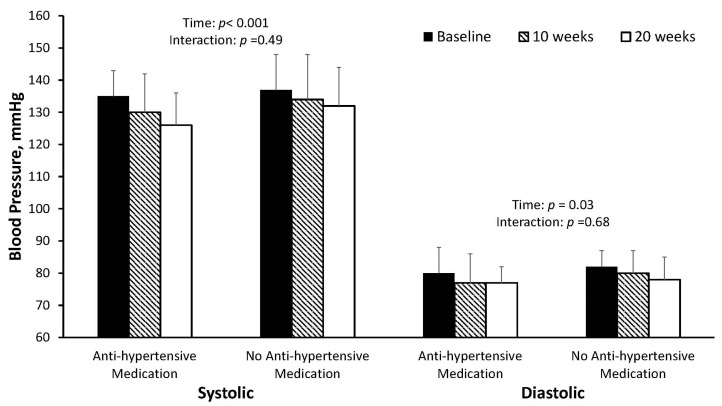
Change in blood pressure in those taking and not taking anti-hypertensive medications. In participants taking anti-hypertensive medications (*n* = 8) at baseline, 10 weeks, and 20 weeks, systolic and diastolic blood pressure decreased from 135 ± 8 to 130 ± 12 to 126 ± 10 mm Hg and 80 ± 8 to 77 ± 9 to 77 ± 5 mm Hg, respectively. In participants not on anti-hypertensive medications (*n* = 13) at baseline, 10 weeks, and 20 weeks, systolic and diastolic blood pressure decreased from 137 ± 11 to 134 ± 14 to 132 ± 12 mm Hg and 82 ± 5 to 80 ± 7 to 78 ± 7 mm Hg, respectively. Error bars indicate standard deviations.

The individual blood pressure responses are presented in Figure 3. Systolic blood pressure decreased from 137 ± 10 mm Hg at baseline to 132 ± 13 mm Hg (*p* = 0.008, g_av_ = 0.41; mean difference from baseline (95% confidence interval [CI]): −4.2 (−1.1, −7.2) mm Hg) at 10 weeks and to 130 ± 11 mm Hg (*p* < 0.001, g_av_ = 0.64; mean difference from baseline (95% CI): −7.0 (−3.9, −10.0) mm Hg) at 20 weeks (overall *p* < 0.001). No significant change in systolic blood pressure was observed from 10 to 20 weeks (*p* = 0.07, g_av_ = 0.16; mean difference (95% CI): −2.8 (0.3, −5.8) mm Hg). Diastolic blood pressure was significantly lower than baseline (81 ± 6 mm Hg) at 20 weeks (77 ± 6 mm Hg, *p* = 0.01, g_av_ = 0.64; mean difference from baseline (95% CI): −3.3 (−1.1, 5.6) mm Hg) but not 10 weeks (79 ± 8 mm Hg, *p* = 0.07, g_av_ = 0.27; mean difference from baseline (95% CI): −2.1 (0.2, −4.3) mm Hg). The change in systolic blood pressure was not correlated with baseline blood pressure at either 10 weeks (*r* = 0.24, *p* = 0.29) or 20 weeks (*r* = −0.08, *p* = 0.74). Further, the reductions in systolic and diastolic blood pressure were not different between those taking anti-hypertensive medications and those who were not taking anti-hypertensive medications (*p* for interaction = 0.49 and 0.68, respectively; Figure 4). Additionally, there was no change in medication (dose or frequency) for any participants throughout the study protocol. There was no significant change in resting heart rate across the 20-week intervention (70 ± 7 bpm at all time points, overall *p* = 0.63, g_av_ = 0.07). All results were similar when only using complete data.

The participants had high overall exercise motivation (5.38 ± 0.73) at baseline, but motivation did not correlate with the change in steps/day observed at 10 (*r* = 0.27, *p* = 0.26) or 20 weeks (*r* = −0.08, *p* = 0.75). At 10 weeks, however, overall exercise action was high (5.34 ± 0.90) and was strongly correlated with the change in steps/day at 10 (*r* = 0.68, *p* = 0.001) and 20 weeks (*r* = 0.53, *p* = 0.02). Overall exercise action at 20 weeks was 5.22 ± 1.09, but it was not correlated with the change in steps/day at 20 weeks (*r* = 0.37, *p* = 0.12). In addition, the participants reported going for a walk becoming more of a habit based on the increase in the SRHI from 3.5 ± 1.2 at baseline to 4.0 ± 1.2 at 10 weeks and 4.2 ± 0.8 at 20 weeks (*p* = 0.01).

## 4. Discussion

In this 20-week e-health walking intervention in sedentary older adults with hypertension, increasing lifestyle walking by approximately 2700 steps/day significantly reduced both systolic and diastolic blood pressure at 20 weeks by 7 and 4 mm Hg, respectively, without significant changes in diet or body weight. Notably, steps/day remained meaningfully greater than baseline values, and blood pressure continued to decline an additional 2 mm Hg even with minimal research personnel contact over the last 10 weeks during the self-maintenance phase. Using an e-health lifestyle walking intervention to meet physical activity guidelines may be an effective means to reduce blood pressure in sedentary older adults with hypertension. 

Our e-health intervention reduced systolic blood pressure, on average, 5 mm Hg after 10 weeks and 7 mm Hg after 20 weeks of the intervention. The reductions in systolic blood pressure from this e-health intervention were similar to reductions in blood pressure reported during lifestyle walking interventions in sedentary older adults with elevated blood pressure both with [33,34] and without [17,35] an e-health component. These studies all suggest that walking to meet the physical activity guidelines is effective for reducing blood pressure in previously sedentary older adults. Nonetheless, it is important to acknowledge that not all lifestyle walking interventions have resulted in significant reductions in blood pressure [18,36,37,38,39,40]. The insignificant changes in blood pressure in prior lifestyle walking interventions may be related to the small changes observed in walking behaviors, greater baseline physical activity participation, minimal research support for obtaining walking goals, or more normal blood pressures observed at baseline. A substantial increase in steps/day to meet the physical activity guidelines may therefore be the key to eliciting favorable changes in blood pressure in sedentary older adults with hypertension. 

The large magnitude of a reduction in systolic blood pressure observed in the present study was similar to or greater than in some previous traditional structured exercise interventions [41,42,43,44,45]. In an exit interview conducted with our participants, 17 of the 18 participants who completed the interviews preferred the lifestyle intervention compared to a structured exercise program that would have occurred in a gym setting. The enjoyment of the lifestyle intervention may partially explain why the blood pressure results were comparable to structured exercise interventions and why individuals continued walking during the second half of the intervention with reduced research personnel assistance. For sedentary older adults with hypertension who are at higher risk for cardiovascular disease, our results suggest that simply increasing walking by at least 2500 steps/day is feasible and beneficial for reducing blood pressure. This simple message can easily be adopted by healthcare professionals for improving the health of their older adults, and it supports lifestyle modification as a critical first step for the treatment of hypertension [2]. 

Steps/day is a simple metric and easy for older adults to interpret and understand, making it an ideal target for promoting physical activity to improve health. Our e-health walking intervention was effective at increasing lifestyle walking in older adults with hypertension. This outcome is evident since the participants met the goal of 3000 additional steps/day 43 ± 14 days (86%) and 40 ± 20 days (80%) at 10 and 20 weeks, respectively, as well as increasing steps/day by 101% (+2760 steps/day) and 77% (+2103 steps/day) at 10 and 20 weeks, respectively, compared to baseline. This large increase in steps/day is at least triple the average increase in steps/day (+790 steps/day) reported by a recent meta-analysis of e-health interventions in older adults, highlighting the effectiveness of our intervention approach and use of the HAPA theory [7]. Previous e-health interventions in older adults, however, have reported variable increases in steps/day. In sedentary mid-aged and older adults (61 ± 6 years old) receiving wearable technology, weekly telephone counseling, and encouragement to achieve 7000 steps/day, Lyons et al. [46] only observed an approximately 20% increase in steps/day (5103 steps/day to 6194 steps/day). In contrast, our results are similar to those reported by Rowley et al. [47], in which walking increased from 4688 to 10,286 steps/day (~119%) after a 12-week intervention in adults 55–80 years of age who received both a pedometer and access to a website for behavior change assistance. The type of e-health intervention (i.e., direct contact, through the web) likely influences the effectiveness of the intervention in a given population and may explain some of the disparate results. E-health interventions, however, can be easily integrated into practice and provide an opportunity for large-scale, remote implementation once determined effective.

All participants received weekly, digital meetings (e.g., video or phone call) with their health coach to help determine strategies to meet their step goals during the first 10 weeks of the active intervention phase. Interestingly, the exercise action score at 10 weeks strongly correlated with the change in steps/day at 10 and 20 weeks, whereas overall motivation at baseline, while high, did not correlate with any changes in steps/day during the intervention. Our results suggest that individuals are more likely to sustain behavior outcomes when there is an emphasis on strategies that develop self-efficacy, such as problem solving and day-to-day action planning skills, rather than relying on initial motivations or intentions, which may be temporary or change over time [48,49]. Ensuring participants are equipped with appropriate planning skills and personalized actionable goals is an important consideration for designing and conducting future physical activity interventions with the goal of high intervention compliance. 

The increase in steps/day and the reductions in blood pressure in the present study both have the potential for substantial benefits regarding risk reduction in sedentary older adults, in addition to the overall health benefits acquired from simply meeting the physical activity guidelines. A recent meta-analysis of 34,584 adults at least 60 years of age suggested that more steps per day lowers mortality risk, with walking 6000–8000 steps/day associated with the lowest overall mortality risk [10]. Likewise, data have suggested a 2 mm Hg reduction in systolic or diastolic blood pressure could lead to a reduction in stroke, coronary heart disease, and overall mortality by at least 6%, 4%, and 3%, respectively [50,51]. Interestingly, the accumulation of aerobic steps, which represents steps achieved during continuous walking bouts of at least 10 min at greater than 60 steps/min, did not influence the change in blood pressure in the present study. We observed a significant increase in aerobic steps at both 10 and 20 weeks, as well as an increase in the number of participants reporting aerobic steps (12, 20, and 17 participants at baseline, 10 weeks, and 20 weeks, respectively). However, at 10 and 20 weeks, there was no significant difference in systolic blood pressure reductions for those increasing aerobic steps by <2000 vs. ≥2000 steps/day from baseline to 10 weeks (−4 vs. −5 mm Hg, *p* = 0.60) or 20 weeks (−6 vs. −9 mm Hg, *p* = 0.42). The results were similar in diastolic blood pressure (both *p* > 0.05 at 10 and 20 weeks). Thus, walking speed and continuous bouts of walking may not be as important as strictly increasing total steps for older adults to reduce blood pressure, similar to results from a previous study [37]. The results of this study are therefore clinically important given the potential translation to lower risk for cardiovascular disease and mortality by simply increasing average lifestyle steps/day in sedentary older adults. 

The primary strength of our study is the use of an e-health intervention that incorporated behavior change techniques from HAPA theory to maximize changes in habitual walking and, due to the e-health nature of the intervention, can be conducted remotely with limited resources (i.e., without a gym, physical contact with participants) and thus implemented on a large scale. Another strength of our study is the objective assessment of physical activity with daily wearing of the pedometer and reporting of steps, as well as the high adherence, which allowed us to report objective physical activity throughout the entire intervention. However, it should be noted that the participants could have tampered with the sealed pedometer, altering the accuracy of the baseline steps. Third, we used a two-phase intervention to observe the ability of older adults to first increase lifestyle walking and then maintain their increased walking with reduced research personnel assistance. A major limitation of this study is that we did not have a control group; thus, our results should be interpreted with caution. Previous studies have suggested no significant changes in walking activity in older adult control groups [17,52]. Second, all steps/day, weight, and blood pressure data were self-reported, although objectively assessed; thus, there is a possibility for some reporting errors or biases. Third, we were unable to test the efficacy of HAPA in this study due to all participants receiving extensive assistance with behavior change techniques. Last, the present study was of a relatively short duration of only 10 weeks with minimal research personnel support; thus, we do not know whether the participants were able to continue their new walking habits and maintain the reductions in blood pressure for a longer duration. Further research is necessary with a larger sample size for greater generalizability, a longer follow-up, and a control group to verify these promising pilot study findings. 

## 5. Conclusions

The results of this pilot e-health lifestyle walking intervention suggest that an extra 3000 steps/day on at least 5 days/week reduced systolic and diastolic blood pressure by 7 and 4 mm Hg, respectively, in sedentary older adults with hypertension. These results could have important implications for healthcare professionals looking for a simple yet effective strategy that can be delivered broadly via e-health technology to reduce blood pressure; however, future large-scale and sufficiently powered randomized, controlled trials are warranted and necessary to verify these results.

## Figures and Tables

**Table 1:** Baseline participant characteristics.

Characteristic	All	Male	Female
*n*	21	8	13
Age, yrs	73 ± 5	73 ± 6	73 ± 5
Height, cm	167.6 ± 8.9	174.5 ± 7.9	162.4 ± 5.1
Weight, kg	85.9 ± 13.9	93.8 ± 12.2	81.1 ± 12.9
BMI, kg/m^2^	30.7 ± 4.2	30.9 ± 4.1	30.7 ± 4.3
Steps, steps/day	3899 ± 2198	4656 ± 2093	3434 ± 2208
Systolic blood pressure, mm Hg	137 ± 10	138 ± 9	136 ± 10
Diastolic blood pressure, mm Hg	81 ± 6	83 ± 7	80 ± 6
Resting heart rate, bpm	70 ± 7	69 ± 6	72 ± 7
Medication usage, *n* (%)			
Anti-hypertensives	8 (39)	3 (38)	5 (38)
Statins	9 (43)	2 (25)	7 (54)

Data are presented as means ± standard deviations or *n* (%). BMI, body mass index. *p* < 0.05 for height and weight between men and women.

**Table 2:** Changes in weight, BMI, and dietary variables throughout lifestyle walking intervention.

Characteristic	Baseline	10 Weeks	20 Weeks	Overall Time Effect *p*-Value	Effect Size ^a^ (Hedges g_av_)
Weight, kg	85.9 ± 13.9	85.1 ± 13.7	84.8 ± 14.9	0.056	0.08
BMI, kg/m^2^	30.6 ± 4.3	30.6 ± 4.2	30.4 ± 4.5	0.051	0.10
Total kilocalories, kcals	2043 ± 640	1816 ± 753	1802 ± 576	0.145	0.29
Protein, g	88 ± 36	77 ± 29	80 ± 32	0.329	0.14
Fat, g	87 ± 28	75 ± 32	75 ± 30	0.126	0.37
Carbohydrates, g	227 ± 89	212 ± 121	204 ± 85	0.569	0.15
Fiber, g	21 ± 8	18 ± 9	19 ± 7	0.641	0.09
Sodium, mg	3331 ± 1133	3519 ± 1353	2961 ± 1143	0.080	0.29

Data are presented as means ± standard deviations. Diet assessed via 24-h recall using ASA24. Analyses adjusted for sex. ^a^ Effect size presented for change from baseline to 20 weeks.

## Data Availability

The datasets used and/or analyzed during the current study are available from the corresponding author on reasonable request.

## Supplementary Materials

The following supporting information can be downloaded at: https://www.mdpi.com/article/10.3390/jcdd10080317/s1, File S1: Exercise Motivation and Action Questionnaires; File S2: Walking Handout; File S3: HAPA Analysis.
