# Supplementary material for: Increasing Lifestyle Walking by 3000 Steps per Day Reduces Blood Pressure in Sedentary Older Adults with Hypertension: Results from an e-Health Pilot Study
Source: J Cardiovasc Dev Dis. 2023 Jul 27;10(8):317. doi: 10.3390/jcdd10080317 (PMC10455876; doi:10.3390/jcdd10080317)
Supplement: Supplementary file 1 [file jcdd-10-00317-s001.zip › Supplementary File 1 - Exercise Motivation and Action Questionnaires.pdf]

Exercise Motivation Question Set

Study ID number: ______________________ Date:_______________

Please answer all questions as honestly as possible. Please note that there are no right or wrong answers and no trick questions. We simply want you to self-reflect on your level of readiness to participate in this study so we can better identify how to help you meet your step-count goals. Your responses will be held in confidence and only used for our research purposes.

1. For you, without participating in regular walking, what is the likelihood that you will suffer from or continue to suffer from high blood pressure in the coming years?

Very unlikely	Quite unlikely	Somewhat unlikely	Neither unlikely nor likely	Somewhat likely	Quite likely	Very likely
☐	☐	☐	☐	☐	☐	☐

2. How confident are you that you will be able to add an extra 3,000 steps to your normal daily routine on 5 days each week?

Not at all confident	Minimally confident	Mildly confident	Moderately confident	Somewhat confident	Quite confident	Very confident
☐	☐	☐	☐	☐	☐	☐

3. If I add an extra 3,000 steps on 5 days each week, then I will feel well-balanced and satisfied.

Completely disagree	Strongly disagree	Disagree	Neither agree nor disagree	Agree	Strongly agree	Completely agree
☐	☐	☐	☐	☐	☐	☐

4. For you, without participating in regular walking, what is the likelihood that you will suffer from or continue to suffer from high blood cholesterol in the coming years?

Very unlikely	Quite unlikely	Somewhat unlikely	Neither unlikely nor likely	Somewhat likely	Quite likely	Very likely
☐	☐	☐	☐	☐	☐	☐

5. If I add an extra 3,000 steps on 5 days each week, then I will do something good for my health and fitness.

Completely disagree	Strongly disagree	Disagree	Neither agree nor disagree	Agree	Strongly agree	Completely agree
☐	☐	☐	☐	☐	☐	☐

6. For you, without participating in regular walking, what is the likelihood that you will suffer from or continue to suffer from cardiovascular disease in the coming years?

Very unlikely	Quite unlikely	Somewhat unlikely	Neither unlikely nor likely	Somewhat likely	Quite likely	Very likely
☐	☐	☐	☐	☐	☐	☐

7. How capable do you see yourself as being able to add an extra 3,000 steps to your normal daily routine on 5 days each week?

Not at all capable	Minimally capable	Mildly capable	Moderately capable	Somewhat capable	Quite capable	Very capable
☐	☐	☐	☐	☐	☐	☐

8. If I add 3,000 steps on 5 days each week, then I will lower my blood pressure.

Completely disagree	Strongly disagree	Disagree	Neither agree nor disagree	Agree	Strongly agree	Completely agree
☐	☐	☐	☐	☐	☐	☐

9. For you, without participating in regular walking, what is the likelihood that you will suffer from or continue to suffer from loss of independence in the coming years?

Very unlikely	Quite unlikely	Somewhat unlikely	Neither unlikely nor likely	Somewhat likely	Quite likely	Very likely
☐	☐	☐	☐	☐	☐	☐

10. How certain are you that you can add an extra 3,000 steps to your normal daily routine on 5 days each week?

Very uncertain	Quite uncertain	Somewhat uncertain	Neither certain nor uncertain	Somewhat certain	Quite certain	Very certain
☐	☐	☐	☐	☐	☐	☐

11. If I add 3,000 steps on 5 days each week, then I will have more energy and feel more alert.

Completely disagree	Strongly disagree	Disagree	Neither agree nor disagree	Agree	Strongly agree	Completely agree
☐	☐	☐	☐	☐	☐	☐

12. I intend to add 3,000 extra steps to my normal daily routine on 5 days each week.

Not at all true	Quite untrue	Somewhat untrue	Neither true nor untrue	Somewhat true	Quite true	Definitely true
☐	☐	☐	☐	☐	☐	☐

Exercise Action Question Set

Study ID number: ______________________ Date:_______________

Please answer all questions as honestly as possible. Please note that there are no right or wrong answers and no trick questions. Your responses will be held in confidence and only used for our research purposes.

1. I have made a detailed plan regarding where to get my extra 3,000 steps.

Not at all true	Quite untrue	Somewhat untrue	Neither true nor untrue	Somewhat true	Quite true	Definitely true
☐	☐	☐	☐	☐	☐	☐

2. I am sure I can keep getting my extra 3,000 steps even if I am tired.

Not at all true	Quite untrue	Somewhat untrue	Neither true nor untrue	Somewhat true	Quite true	Definitely true
☐	☐	☐	☐	☐	☐	☐

3. I have made a detailed plan regarding on what days of the week to get my extra 3,000 steps.

Not at all true	Quite untrue	Somewhat untrue	Neither true nor untrue	Somewhat true	Quite true	Definitely true
☐	☐	☐	☐	☐	☐	☐

4. I am confident I can keep getting my extra 3,000 steps even if I have too much work to do.

Completely disagree	Strongly disagree	Disagree	Neither agree nor disagree	Agree	Strongly agree	Completely agree
☐	☐	☐	☐	☐	☐	☐

5. I have made a detailed plan regarding what time(s) of day to get my extra 3,000 steps.

Not at all true	Quite untrue	Somewhat untrue	Neither true nor untrue	Somewhat true	Quite true	Definitely true
☐	☐	☐	☐	☐	☐	☐

6. I have made a detailed plan regarding where to get my extra 3,000 steps if there is bad weather.

Not at all true	Quite untrue	Somewhat untrue	Neither true nor untrue	Somewhat true	Quite true	Definitely true
☐	☐	☐	☐	☐	☐	☐

7. I am sure I can resume getting my extra 3,000 steps even if I have interrupted my routine more than once.

Completely disagree	Strongly disagree	Disagree	Neither agree nor disagree	Agree	Strongly agree	Completely agree
☐	☐	☐	☐	☐	☐	☐

8. I have made a detailed plan regarding how to get my extra 3,000 steps if something unexpected comes up.

Not at all true	Quite untrue	Somewhat untrue	Neither true nor untrue	Somewhat true	Quite true	Definitely true
☐	☐	☐	☐	☐	☐	☐

9. I am able to keep getting my extra 3,000 steps even if I am feeling down or depressed.

Completely disagree	Strongly disagree	Disagree	Neither agree nor disagree	Agree	Strongly agree	Completely agree
☐	☐	☐	☐	☐	☐	☐

10. I have made a detailed plan regarding when to get my extra 3,000 steps if I miss a day.

Not at all true	Quite untrue	Somewhat untrue	Neither true nor untrue	Somewhat true	Quite true	Definitely true
☐	☐	☐	☐	☐	☐	☐

11. I am certain I can keep getting my extra 3,000 steps <u>even if there are other more interesting things to do.</u>

Completely disagree	Strongly disagree	Disagree	Neither agree nor disagree	Agree	Strongly agree	Completely agree
☐	☐	☐	☐	☐	☐	☐

12. I am confident I can <u>resume</u> getting my extra 3,000 steps <u>even if I weren't physically active for a period of time.</u>

Completely disagree	Strongly disagree	Disagree	Neither agree nor disagree	Agree	Strongly agree	Completely agree
☐	☐	☐	☐	☐	☐	☐
