# Supplementary material for: Increasing Lifestyle Walking by 3000 Steps per Day Reduces Blood Pressure in Sedentary Older Adults with Hypertension: Results from an e-Health Pilot Study
Source: J Cardiovasc Dev Dis. 2023 Jul 27;10(8):317. doi: 10.3390/jcdd10080317 (PMC10455876; doi:10.3390/jcdd10080317)
Supplement: Supplementary file 1 [file jcdd-10-00317-s001.zip › Supplementary File 2 - Walking Handout.pdf]

The 3Ps of the 3K study

*"Inspiration is what gets you started. **Habit** is what keeps you going!"*

Plan

Walk whenever you can:

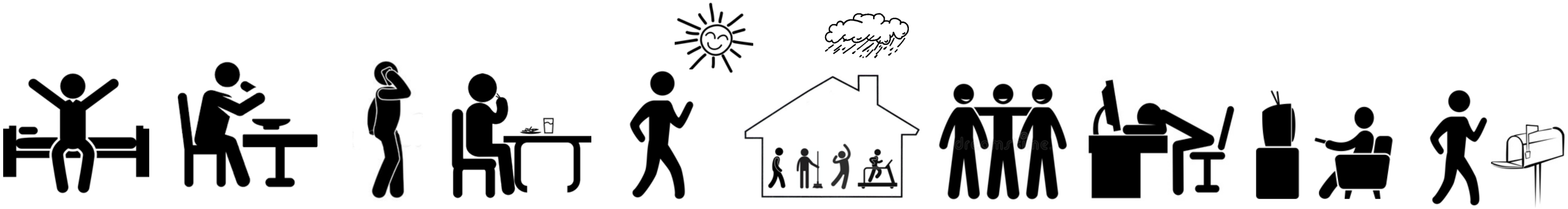

First thing in the morning | After your breakfast | While on the phone | After your lunch | When it's nice outside | When it's **not** nice outside | When you're with friends | When you're bored | During commercials | When you have mail

Place

Walk wherever there is space:

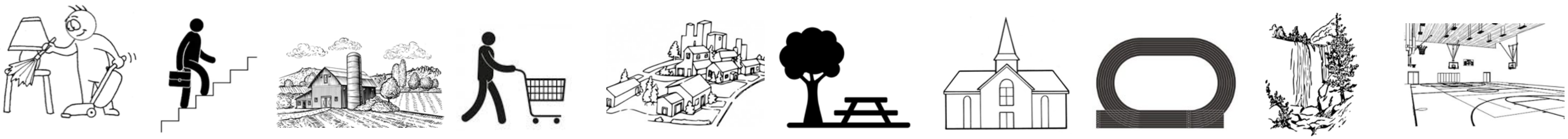

Home | Work | Farm | Supermarket | Neighborhood | Local park | Place of worship | Local track | State park | Recreation center

Play

Find ways to make walking meaningful to you:

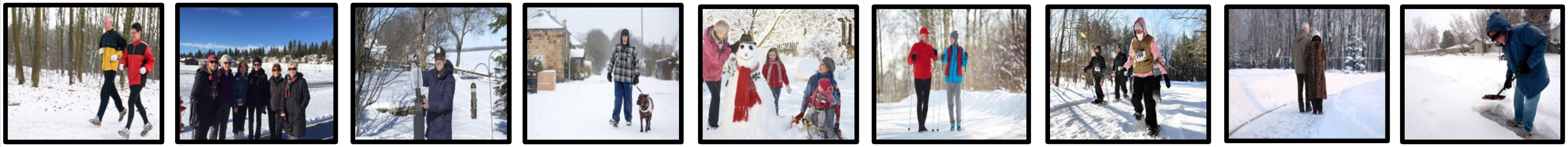

Walk for your health | Walk for friendship | Walk in the garden | Walk the dog | Walk with your family | Walk to explore nature | Walk for charity | Walk to clear your mind | Walk to do tasks

Physical Activity Epidemiology Laboratory
