# Supplementary material for: Increasing Lifestyle Walking by 3000 Steps per Day Reduces Blood Pressure in Sedentary Older Adults with Hypertension: Results from an e-Health Pilot Study
Source: J Cardiovasc Dev Dis. 2023 Jul 27;10(8):317. doi: 10.3390/jcdd10080317 (PMC10455876; doi:10.3390/jcdd10080317)
Supplement: Supplementary file 1 [file jcdd-10-00317-s001.zip › Supplementary File 3 - HAPA analysis.pdf]

Baseline Participant Characteristics by HAPA group

Characteristic	All	Control	HAPA	p-value
n	21	11	10	
Age, yrs	73 ± 5	73 ± 5	74 ± 5	0.67
Female, n (%)	13 (62)	6 (55)	7 (70)	0.47
Height, cm	167.6 ± 8.9	166.6 ± 9.0	167.5 ± 8.5	0.83
Weight, kg	85.9 ± 13.9	85.3 ± 8.4	86.6 ± 18.7	0.83
BMI, kg/m^2	30.7 ± 4.2	30.8 ± 3.5	30.7 ± 5.0	0.94
Steps, steps/day	3899 ± 2198	4194 ± 2371	3575 ± 2064	0.53
Systolic blood pressure, mmHg	137 ± 10	136 ± 12	137 ± 7	0.76
Diastolic blood pressure, mmHg	81 ± 6	80 ± 7	82 ± 5	0.40
Resting heart rate, bpm	70 ± 7	69 ± 6	72 ± 7	0.29
Medication Usage, n (%)				
Anti-hypertensive	8 (39)	5 (45)	3 (30)	0.46
Statin	9 (43)	5 (45)	4 (40)	0.80

Data presented mean ± standard deviation or n (%). BMI, body mass index; HAPA, Health Action Process Approach.

Change in steps, systolic, and diastolic blood pressure across intervention by HAPA group.

					p-value		
		Baseline	10 Weeks	20 Weeks	Group	Time	GroupxTime Interaction
Steps, steps/day					0.59	<0.001	0.58
	Control	4194 ± 2371	6852 ± 3103	5836 ± 2684			
	HAPA	3575 ± 2064	6179 ± 2179	5879 ± 2062			
Systolic Blood Pressure, mmHg					0.99	<0.001	0.73
	Control	136 ± 12	133 ± 13	130 ± 13			
	HAPA	137 ± 7	132 ± 13	129 ±10			
Diastolic Blood Pressure, mmHg					0.43	0.02	0.55
	Control	80 ± 7	77 ± 8	77 ± 5			
	HAPA	82 ± 5	81 ± 7	78 ± 8			

Data presented mean ± standard deviation
